# Supplementary material for: Evidence supported by Mendelian randomization: impact on inflammatory factors in knee osteoarthritis
Source: Front Med (Lausanne). 2024 May 28;11:1382836. doi: 10.3389/fmed.2024.1382836 (PMC11165061; doi:10.3389/fmed.2024.1382836)
Supplement: Supplementary file 1 [file Data_Sheet_1.ZIP › data/GCST90274818_5e6/818.forest.pdf]

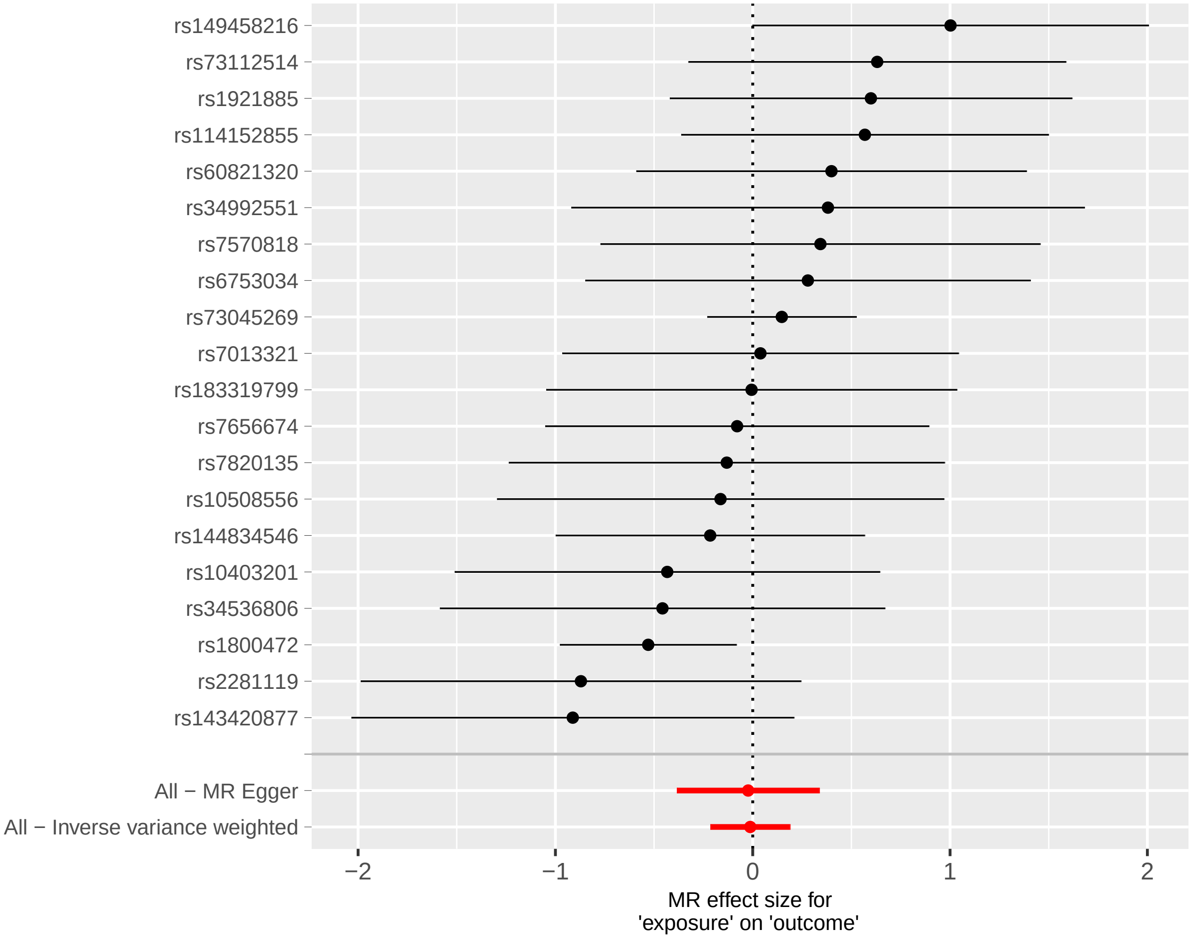

rs149458216
rs73112514
rs1921885
rs114152855
rs60821320
rs34992551
rs7570818
rs6753034
rs73045269
rs7013321
rs183319799
rs7656674
rs7820135
rs10508556
rs144834546
rs10403201
rs34536806
rs1800472
rs2281119
rs143420877
All − MR Egger
All − Inverse variance weighted
−2
−1
0
1
2
MR effect size for
'exposure' on 'outcome'
